# Low-cost monocular RGB-based 3D structural mapping for horticultural plants via semantic scene completion

**DOI:** 10.3389/fpls.2026.1825547

**Published:** 2026-07-22

**Authors:** Ruijun Jing, Rui An, Zhuoxing Li, Jiliang Mu, Jing Liu, Zhiguo Zhao, Ruiyan Ma, Xiujian Chou

**Affiliations:** 1 School of Instrument and Electronics, North University of China, Taiyuan, China; 2 School of Software, Shanxi Agricultural University, Taiyuan, China; 3 College of Plant Protection, Shanxi Agricultural University, Taiyuan, China; 4 Shanxi Key Laboratory of Ferroelectric Physical Micro-nano Devices and Systems, North University of China, Taiyuan, China; 5 School of Automation and Software Engineering, Shanxi University, Taiyuan, China

**Keywords:** depth-aware decoder module, geometry encoder, monocular perception, semantic scene completion, structural mapping

## Abstract

Precision agriculture increasingly relies on detailed structural information, such as canopy height and canopy volume, to enhance crop health monitoring and operational safety. However, existing methods based on costly LiDAR or RGB-D sensors are often impractical for large-scale deployment in dynamic and unstructured horticultural environments. Furthermore, conventional 2D segmentation and SLAM-based pipelines typically generate sparse, geometrically inconsistent semantic maps which are insufficient for actionable structural analysis in agricultural applications. To overcome these limitations, we propose a monocular 3D structural mapping framework tailored for horticultural plants via semantic scene completion. At inference, the proposed model takes a single RGB image as input and predicts voxel-wise geometry and semantics, from which task-oriented structural maps, including canopy height, canopy volume, and obstacle-aware traversability layers, are derived. Specifically, we first introduce a Depth-Aware Decoder Module that explicitly recovers depth in the spatial domain and fuses 2D-to-3D features, thereby mitigating depth ambiguity and reducing reliance on accurate pose. Second, an NCS-Guided Geometry Encoder is designed to inject normalized depth into voxel positional embeddings, enabling self-attention to perform global relational modeling within a depth-aware geometric coordinate system. In addition, a Global Encoder is utilized to refine local structural details, while an occupancy head produces the final 3D semantic completion outputs. We construct a horticultural 3D semantic scene dataset using an RGB-D sensor, which serves as a benchmark for evaluating our method, while the deployed model remains RGB-only. Extensive quantitative and qualitative experiments are conducted on both the Semantic-KITTI dataset and our dataset. On our dataset, the method achieves 82.31% occupancy IoU, 84.26% mIoU, and 86.25% precision. Beyond voxel-level evaluation, manual field measurements further show canopy height MAE values of 0.019-0.026 m and canopy volume proxy relative errors of 8.4%-11.4%. These results demonstrate the effectiveness of our approach in real-world agricultural scenarios, providing actionable structural insights for crop monitoring and autonomous robotic operations.

## 1 Introduction

Driven by rapid progress of artificial intelligence and robotics, precision horticulture is transitioning from plot-level management toward plant-level, data-driven operations, including canopy growth monitoring (Bai et al., 2023), variable-rate field practices (Jing et al., 2025), and autonomous inter-row operation (Xiao et al., 2023). These tasks require not only recognizing crops and obstacles, but also producing actionable and spatially consistent 3D structural understanding that can be directly consumed by agronomic assessment and robotic decision-making. In practice, actionable horticultural mapping should provide interpretable structural layers such as canopy height distribution, volumetric canopy density, and obstacle-aware traversable regions, which directly support agronomic assessments and enhance operational safety. However, existing mapping systems often fail to provide the dense and agronomically interpretable structural representations required for canopy monitoring and robotic navigation.

In controlled-environment agriculture, factors such as reflective materials and illumination variations further complicate depth estimation and structural reconstruction (Ruijun and JingKai, 2024). Although LiDAR and RGB-D sensors can provide direct depth measurements, their inherent system costs, power consumption, and deployment complexity render them impractical for large-scale horticultural applications. Previous studies have explored SLAM-based 3D mapping for agricultural environments, including monocular SLAM (Sukvichai et al., 2023; Chen et al., 2024) or RGB-D SLAM (Su et al., 2024), but these approaches often produce sparse, surface-centric maps and require precise pose estimation. To enrich semantics, several works integrate 2D semantic segmentation with SLAM or 3D reconstruction (Lv et al., 2024; Liu et al., 2025a) by projecting 2D labels onto point clouds or reconstructed surfaces. However, most of these methods follow a pipeline-style (Xiong et al., 2023; Pan et al., 2024; Liu et al., 2025b; Wang et al., 2025) design where geometry reconstruction and semantic inference are performed independently. Consequently, these methods still yield geometrically misaligned and temporally unstable representations.

To address these limitations, we formulate low-cost horticultural structural perception as a monocular RGB-based semantic scene completion (SSC) problem (Cao and De Charette, 2022; Roldão et al., 2022). Unlike existing monocular SSC methods, our goal is not only to reconstruct semantic occupancy, but also to transform completed 3D plant structures into task-oriented maps that can support crop monitoring and robotic operation. Unlike point-based maps, SSC provides a dense volumetric representation that jointly encodes both geometry and semantics and can explicitly reason about occluded or unobserved spaces. These characteristics are particularly beneficial for horticultural applications, where canopy occlusion is common and structural cues are often incomplete when viewed from a single perspective.

Most existing SSC methods are developed and evaluated primarily on urban driving or indoor benchmarks (Mei et al., 2024; Lu et al., 2025; Tseng et al., 2025) and often rely on expensive multi-sensor setups or accurate pose estimation, making them difficult to directly apply to horticultural environments. Moreover, existing monocular SSC methods focus on urban semantic reconstruction and do not produce the task-level structural maps required for agronomic decision-making. Similarly, LiDAR simulation methods for adverse weather (Liu et al., 2026) and multi-robot collaborative 3D measurement systems (Zhang et al., 2026) achieve high-fidelity reconstruction but are either sensor-intensive or complex to deploy. Recent studies on robust 3D perception and collaborative 3D measurement highlight the importance of reliable structural reasoning for engineering deployment (Gan et al., 2022; Su et al., 2026). However, these methods are generally designed for non-agricultural scenarios or depend on multi-sensor configurations (Xu et al., 2026), thus failing to directly address low-cost monocular plant structural mapping.

To overcome these problems, we develop a novel monocular 3D structural mapping framework for horticulture via SSC. At the deployment stage, the proposed framework uses only a single RGB image as input to reconstruct dense voxel-wise semantic occupancy and derive task-oriented structural maps, including canopy height, canopy volume, and obstacle-aware traversability layers. During dataset construction, RGB-D observations are used only to generate reliable voxel-wise ground truth for supervised training. This enables the model to learn 3D structural priors from depth-supported supervision while maintaining low-cost RGB-only inference. Our approach not only reduces the cost and complexity associated with traditional sensor-based systems but also provides a practical and scalable solution for real-world agricultural environments.

The main contributions of this work are summarized as follows:

1. Task Formulation for Horticultural Structural Mapping. We formulate low-cost horticultural structural perception as a monocular RGB-based semantic scene completion problem. We derive agronomically interpretable structural maps, including canopy height, a canopy volume proxy, and obstacle-aware traversability, from the completed semantic voxel grids.
2. Depth-Aware Decoder Module (DADM). We propose a depth-aware monocular SSC framework that embeds structured geometric priors into the 2D-to-3D representation learning process. The proposed Depth-Aware Decoder Module explicitly reconstructs depth-aware voxel representations from monocular image features by combining depth probability distributions with progressive NCS-based voxel refinement, thereby reducing monocular depth ambiguity and improving geometric consistency.
3. NCS-Guided Geometry Encoder (NGE). We introduce an NCS-Guided Geometry Encoder that incorporates normalized depth cues into voxel positional embeddings, allowing deformable attention and self-attention to perform geometric-semantic reasoning in a depth-aware coordinate system.
4. Horticultural SSC Dataset. We construct a dedicated dataset following the Semantic-KITTI annotation protocol to benchmark monocular SSC in agricultural scenarios.

Collectively, these designs shift monocular SSC from generic urban-scene semantic reconstruction toward task-oriented horticultural structural mapping. Our framework differs from existing SSC methods not only in network design, but also in its target output, dataset construction, and field-level structural validation. By integrating depth-aware priors with geometry-semantic reasoning and producing actionable task-level maps, our method bridges the gap between urban/indoor SSC models and real-world horticultural applications, addressing monocular depth ambiguity, weak-pose dependency, and the need for interpretable structural outputs.

## 2 Methods

**Figure 1:**
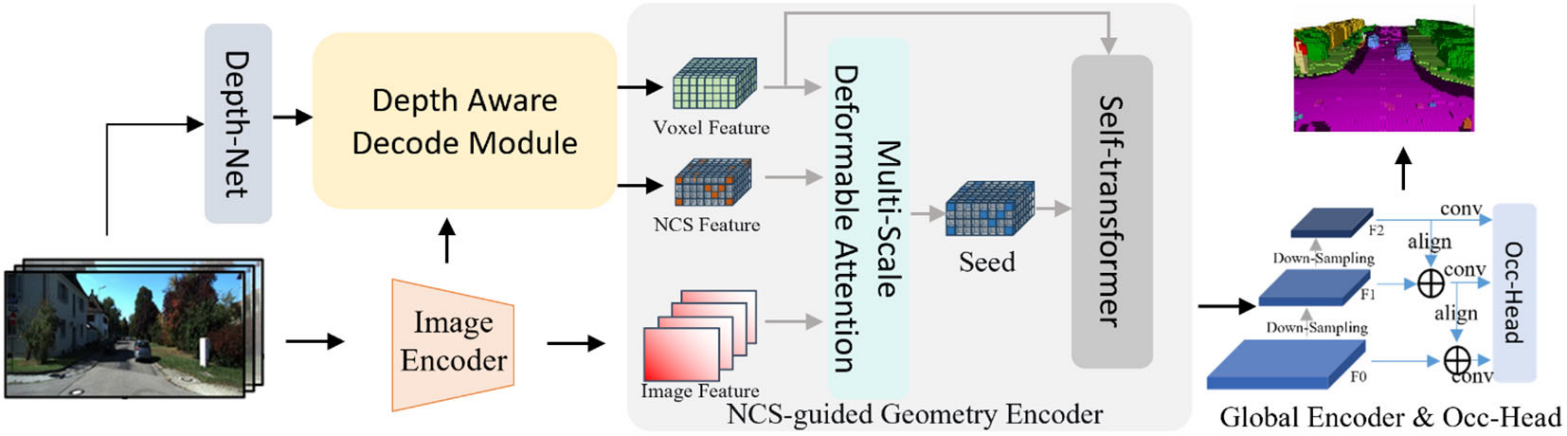
The framework of monocular semantic scene completion.

**Figure 1** illustrates the proposed monocular 3D structural mapping framework. Our framework is designed as an RGB-only inference model for low-cost horticultural deployment. Specifically, during inference, the model takes a single RGB image as input and directly predicts a 3D semantic voxel representation. Our approach enables actionable structural analysis and decision-making for agronomic tasks without the need for additional trainable modules, which distinguishes it from conventional methods that often rely on expensive sensors or multi-stage processing.

The proposed architecture consists of three core components: the Depth-Aware Decoder Module (DADM), the NCS-Guided Geometry Encoder (NGE), and the Global Encoder with an occupancy head (Occ-Head).

To address the high variability and complexity inherent in horticultural environments, we introduce probabilistic guidance that enhances the model’s robustness in uncertain and dynamic field scenarios. This probabilistic approach facilitates more accurate depth recovery and semantic inference, which are critical for robust 3D mapping in cluttered environments. Specifically, we propose the Depth-Aware Decoder Module (DADM), which couples explicit depth recovery with 2D-to-3D branch feature fusion, effectively alleviating depth projection ambiguity and reducing the system’s dependence on accurate sensor pose parameters. Starting from high-resolution 2D encoded features, the system explicitly reconstructs 3D depth voxels in the Normalized Coordinate Space (NCS) (Shi et al., 2020). Through geometrically consistent view transformations and voxel pooling, it generates 3D representations on the target voxel grid, where a Geometry-Aware Transformer (Yu et al., 2024a) is applied to model global semantic and geometric relationships (Yu et al., 2024b). Finally, the Global Encoder and Occ-Head module are employed to generate the final 3D semantic structural maps. From this voxel representation, canopy height and volume layers are computed via column-wise occupancy statistics, while traversability is estimated by combining obstacle occupancy and canopy intrusion into a free-space layer.

It should be noted that RGB-D data are used only during dataset construction and supervised training, not during final inference. We separate the data-generation stage from the deployment stage. The RGB-D sensor is used to build the voxel-level ground truth of Horticultural SSC Dataset. The depth observations, together with semantic point-cloud annotations, are used to generate voxel-wise ground truth for geometry and semantics. These voxel labels provide the supervision required for training the monocular SSC model. After training, the network has learned depth-aware 3D structural priors from the voxel-level ground truth and can therefore infer 3D structural maps from a single RGB image without requiring depth input, LiDAR, or RGB-D sensing during deployment.

### 2.1 Depth-aware decoder module

**Figure 2:**
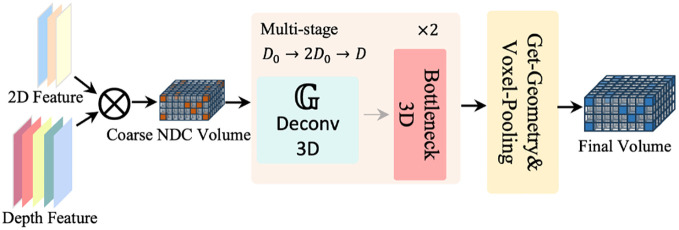
The framework of depth-aware decoder module.

Conventional methods that project image features into initial voxel representations often rely heavily on accurate sensor pose information, as seen in techniques like Lift-Splat-Shoot (LSS) (Cao and De Charette, 2022). However, this single-step lifting approach does not explicitly recover 3D spatial structures, resulting in significant depth ambiguity and feature-scale uncertainty. These limitations propagate noise into subsequent 3D Transformers, thereby impairing structural consistency and accuracy in agricultural applications. To address depth ambiguity and improve feature alignment, we propose the Depth-Aware Decoder Module, which combines the concept of normalized coordinates with probabilistic guidance to explicitly recover depth and fuse 2D-to-3D features. This module enables more accurate depth reconstruction and provides rich spatial context necessary for effectively modeling agricultural environments. The overall architecture is illustrated in **Figure 2**.

The DADM constructs coarse-grained features in the Normalized Coordinate Space (NCS) based on depth probability distributions. Multi-level 3D transposed convolution layers are then used to progressively recover the depth resolution of these features, expanding them along the depth dimension until the target depth resolution (D) is reached.

Given the 2D encoded features ${\text{X}}^{2\text{D}}\in {\mathbb{R}}^{\text{C}\times \text{H}\times \text{W}}$ and the corresponding depth probability volume, we project the 2D features into an intermediate embedding space with channels *C_m_*, which leverages depth-guided probabilistic feature aggregation to enhance both spatial resolution and depth accuracy.

Performing the lifting operation over D depth layers incurs a high computational cost. To control complexity while preserving structural expressiveness, we first down-sample D along the depth dimension to obtain a coarse depth resolution D_0_ = D/s, as expressed in Equation 1.

(1) $$\tilde{D}\left(d_{0},h,w\right)=P_{s}\left(D\left(:,h,w\right)\right),\;d_{0}=1,\ldots ,D_{0}$$

where P_s_(·) denotes max-pooling along the depth dimension. We then perform probabilistic aggregation at the coarse depth resolution to obtain a coarse voxel volume ${\text{V}}_{0}\in {\mathbb{R}}^{{\text{C}}_{\text{m}}\times {\text{D}}_{0}\times \text{H}\times \text{W}}$, as defined in Equation 2.

(2) $$V_{0}\left(c,d_{0},h,w\right)=X^{proj}\left(c,h,w\right)\tilde{D}\left(d_{0},h,w\right)$$

In this manner, an initial voxel representation V_0_ in the NCS is constructed. By stacking transposed convolution and Bottleneck3D modules, a coarse-to-fine refinement of depth information is progressively achieved. Although transposed convolutions are used to recover NCS feature representations, they lack sufficient semantic and geometric relational modeling. The Bottleneck3D modules are therefore introduced to capture 3D spatial understanding and geometric structures within the depth scale, thereby enhancing the overall representational capacity of the features. This refinement process is formulated in Equation 3.

(3) $$V_{s+1}={ℬ}_{s}\left(U_{s}\left(V_{s}\right)\right),\;s=0,\ldots ,S-1$$

Here $U_{s}\left(\cdot \right)$ denotes a 3D transposed convolution operator that increases the resolution along the depth dimension. ${ℬ}_{s}\left(\cdot \right)$ represents a residual 3D Bottleneck module that models local geometric and semantic relationships. The architecture of the 3D Bottleneck is defined in **Equations 4** and **5**:

(4) $$\begin{array}{c} z_{1}=\sigma \left(conv_{1\times 1\times 1}\left(X\right)\right)\\ z_{2}=\sigma \left(conv_{3\times 3\times 3}\left(z_{1}\right)\right)\\ z_{3}=BN\left(\sigma \left(conv_{1\times 1\times 1}\left(z_{2}\right)\right)\right) \end{array}$$

(5) $$X^{NCS}=\sigma \left(z_{3}+down\left(X\right)\right)$$

where σ denotes the ReLU activation. $conv_{1\times 1\times 1}$ is used for channel projection, while the middle $conv_{3\times 3\times 3}$ explicitly aggregates neighboring voxel features and captures local 3D geometric context. *down*(·) denotes either an identity mapping or a 1×1×1 projection used to match the channel dimension of the residual branch.

The 3D Bottleneck blocks perform convolutions on a regular NCS grid, thereby avoiding the inconsistency of convolutional receptive fields caused by camera poses in the world coordinate system. This design provides geometrically consistent and less sensitive to pose variations 3D representations for subsequent modules. After *S* stages, an NCS voxel representation with a depth resolution consistent with the target depth is obtained, as defined in Equation 6.

(6) $$X^{NCS}=V_{S}\in {\mathbb{R}}^{C_{0}\times D\times H\times W}$$

Finally, *X^NCS^* is mapped into the target voxel space via a geometric projection function $\text{Π}∶S^{N}\to S^{T}$ followed by a voxel pooling operation, yielding $S^{T}\in {\mathbb{R}}^{C_{0}\times X\times Y\times Z}$, as defined in Equation 7.

(7) $$X^{T}=Pool\left(\text{Π}\left(X^{NCS}\right)\right)$$

### 2.2 NCS-guided geometry encoder

**Figure 3:**
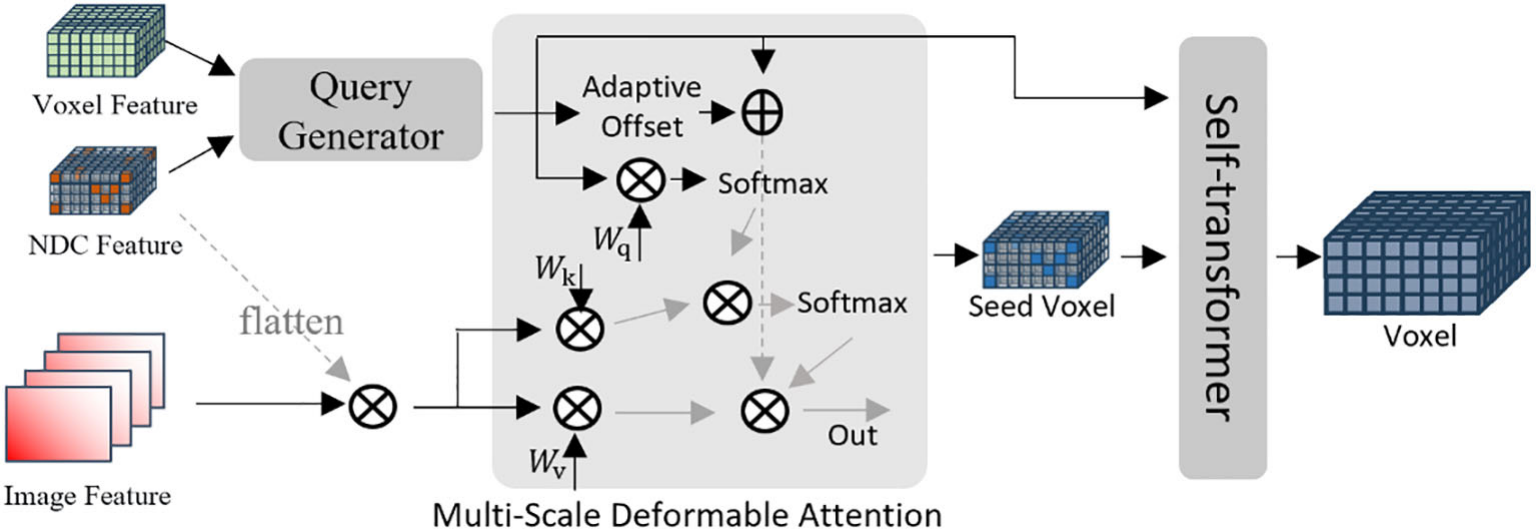
NCS-guided geometry encoder.

Conventional Transformer-based models often rely on conventional (x, y, z) 3D coordinates for positional encoding (Wang and Tong, 2024). This reliance leads to misaligned semantic reasoning and inaccurate structural predictions, particularly in dynamic and cluttered agricultural environments. To resolve this limitation, we propose NCS-Guided Geometry Encoding (NGE), which integrates normalized depth into voxel positional embeddings, enabling that all attention operations are performed within a depth-aware geometric coordinate system. The depth-guided encoding enables the model to more effectively reason about global geometric relationships and handle occlusions in agricultural scenes. The overall architecture of NGE is illustrated in **Figure 3**.

#### 2.2.1 Query generator

The feature volume (Zhang et al., 2023) in the target voxel space S^T^ is denoted as ${\text{V}}^{\text{T}}\in {\mathbb{R}}^{\text{B}\times \text{C}\times \text{X}\times \text{Y}\times \text{Z}}$. Let $\left(N_{x},N_{y},N_{z}\right)$ denote the voxel grid dimensions. The corresponding geometric center coordinates of each voxel are given by Equation 8:

(8) $$p_{i,j,k}^{T}=\left(x_{i,j,k}^{T},y_{i,j,k}^{T},z_{i,j,k}^{T}\right),\;i\in [0,\;X),j\in [0,\;Y),k\in [0,\;Z)$$

We first obtain the NCS depth voxel representation, as defined in Equation 9.

(9) $$D^{NCS}\in {\mathbb{R}}^{B\times 1\times X\times Y\times Z}$$

To enable a unified representation, we combine the (x, y, z) coordinates with the NCS depth (Mei et al., 2024) to construct a 4D geometric vector for each voxel, as defined in Equation 10.

(10) $$g_{i,j,k}=\left[x_{i,j,k}^{T},y_{i,j,k}^{T},z_{i,j,k}^{T},d_{i,j,k}^{NCS}\right]$$

Where $d_{i,j,k}^{NCS}$ denotes the normalized depth of the corresponding voxel in the NCS. As a result, each voxel is defined in a 4D geometric space, and the 4D geometric space is flattened to yield, as defined in Equation 11.

(11) $$G={\left\{g_{n}\right\}}_{n=1}^{N_{v}},N_{v}=X\cdot Y\cdot Z$$

Where *n* denotes the voxel index. We design a lightweight NCS-aware positional encoder to implicitly encode the 4D geometric cues into compact spatial embeddings, as defined in Equation 12.

(12) $$PE^{NCS}\left(g_{n}\right)=\phi \left(g_{n}\right)=W_{2}\sigma \left(W_{1}g_{n}+b_{1}\right)+b_{2}$$

where σ(·) denotes the ReLU activation. $W_{1}\in {\mathbb{R}}^{d\times 4}$ and $b_{1}\in {\mathbb{R}}^{d}$ denote the learnable parameters of the first linear layer, while $W_{2}\in {\mathbb{R}}^{D\times d}$ and $b_{2}\in {\mathbb{R}}^{D}$ denote the learnable parameters of the second linear layer. Here, *d* is the hidden dimension of the positional encoder and *D* is the channel dimension of the voxel feature. For each voxel, the NCS-aware positional embedding is obtained as $e_{n}^{NCS}=PE^{NCS}\left(g_{n}\right)\in {\mathbb{R}}^{D}$, $E^{NCS}={\left\{e_{n}^{NCS}\right\}}_{n=1}^{N_{v}}$.

This mechanism explicitly integrates normalized depth information by embedding depth cues into the spatial positions. The NCS-aware geometric encoding is then added to the voxel queries, as shown in Equation 13.

(13) $${\tilde{q}}_{n}=q_{n}^{base}+e_{n}^{NCS}$$

#### 2.2.2 Deformable transformer encoder

While image features preserve high-resolution appearance cues, voxel queries are essential for capturing 3D structural awareness. Fusing these two representations requires addressing two challenges: (1) a voxel’s 3D location cannot be directly and accurately mapped onto the 2D image space due to depth ambiguity; (2) significant geometric variations across camera viewpoints make fixed receptive fields prone to severe blurring. To overcome these problems, we propose the Deformable Transformer Encoder, which performs voxel initialization via depth-guided deformable sampling.

Voxels with similar spatial coordinates (*x*, *y*, *z*) but markedly different depths are thus separated in the NCS-aware positional embedding space, which alleviates the feature-depth ambiguity observed in conventional FLoSP/LSS lifting. Building upon this, we construct an attention module that operates on Equation 14.

(14) $$Q=\tilde{Q}W_{Q},K=FK_{I},V=FV_{I}$$

where *F* denotes the flattened voxel features, with $f_{n}\in {\mathbb{R}}^{D},F={\left\{f_{n}\right\}}_{n=1}^{N_{v}}$. By enhancing the geometric prior of the Query, the attention (Li et al., 2026) becomes more sensitive to variations along the depth dimension.

Let *K_C_*, *R_c_* and *t* be the intrinsic matrix, rotation matrix, and translation vector of the *c*-th camera, respectively, and let l index the feature pyramid level. The projection of the -th voxel center onto the -th camera is defined in Equation 15.

(15) $${\tilde{p}}_{i,c}^{l}=K_{c}\left(R_{c}r_{i}+t\right)$$

Here, each voxel query corresponds to an NCS voxel center $r_{i}=\left(x_{i},y_{i},z_{i}\right)$. After normalization, the feature map coordinates are given, where $s_{w}^{\;l}$ and $s_{h}^{\;l}$ denote the downsampling stride of the *l*-th feature level along the width and height, respectively.

Because the projected points may not fall on the target object. We therefore introduce learnable offsets to generate adaptive sampling locations around the base projection. Specifically, each query *q_i_* predicts multi-scale, multi-point offsets via a linear layer, as defined in Equation 16.

(16) $$\Delta p_{i,l,p}=W_{offset}q_{i}+b_{offset}$$

where *q_i_* is the voxel query. The sampling locations are computed as ${\tilde{p}}_{i,c,p}^{l}={\tilde{p}}_{i,c}^{l}+\Delta p_{i,l,p}$We further incorporate the image feature values and the image-wise depth distribution *D* to predict attention weights: $W_{i,l,n,p}=softmax\left({\left(W_{q}q_{i}\right)}^{T}W_{k}v_{i,l,n,p}\right)$ where *v_i_*,*_l_*,*_n_*,*_p_* denotes the sampled image feature at location ${\tilde{p}}_{\text{i,c,p}}^{\text{l}}$. Finally, multi-scale and multi-point aggregation across all cameras yields the seed voxel feature: $f_{i}^{seed}=\sum_{c=1}^{N_{cam}}\sum_{l=1}^{L}\sum_{p=1}^{P}W_{i,l,n,p}\cdot W_{v}V_{c}^{l}{\tilde{p}}_{i,c,p}^{l}$.

#### 2.2.3 Multi-head self-attention

After obtaining the initial voxel observations from the input image, further spatial reasoning in the 3D voxel space is required to fill occluded regions, enhance geometric consistency, and establish global semantic relations. To this end, we employ a multi-head self-attention module to fuse the seed voxel features with voxel queries (Q). This enables the modeling of long-range dependencies within the voxel grid, allowing semantic scene completion to preserve structural coherence even under severe occlusion.

To provide geometric priors, we transform the normalized 3D coordinates of the seed voxel centers $r_{i}\in {\left[0,1\right]}^{3}$ into positional embeddings, as defined in Equation 17.

(17) $${\tilde{q}}_{i}=q_{i}+PE\left(r_{i}\right)$$

For level *l*, the queries, keys, and values are defined as $Q^{\left(l\right)}=\tilde{Q}W_{Q},K^{\left(l\right)}=\tilde{Q}W_{K},V^{\left(l\right)}=\tilde{Q}W_{V}$, $Q^{\left(l\right)}=\left[q_{1}^{\left(l\right)},q_{2}^{\left(l\right)},\ldots ,q_{N_{v}}^{\left(l\right)}\right]$. The attention distribution is defined as Equation 18.

(18) $$a_{ij}^{\left(l\right)}=\frac{exp\left(\frac{{\left(q_{i}^{\left(l\right)}\right)}^{T}k_{j}^{\left(l\right)}}{\sqrt{C}}\right)}{\sum_{k=1}^{N_{v}}exp\left(\frac{{\left(q_{i}^{\left(l\right)}\right)}^{T}k_{k}^{\left(l\right)}}{\sqrt{C}}\right)}$$

The updated output is computed as $q_{i}^{\left(l+1\right)}=\sum_{j=1}^{N_{v}}a_{ij}^{\left(l\right)}v_{j}^{\left(l\right)}$

### 2.3 Global encoder and occ-head

Agricultural scenes exhibit highly complex 3D structures, characterized by abundant non-rigid elements, irregular geometry, and substantial local structural variations. These complexities make it difficult to provide consistent and accurate representations for tasks such as crop monitoring and robotic navigation. To improve the network’s sensitivity to fine-grained local geometry and voxel-level details, we employ a Global Encoder to refine voxel-space representations, and an Occ-Head to produce the final 3D semantic scene completion outputs.

The Global Encoder (Miao et al., 2023) enhances the structural consistency and spatial fidelity of the voxel representation through hierarchical 3D convolutional encoding, and a multi-scale voxel feature pyramid. It consists of two tightly coupled components: a 3D convolutional backbone that extracts hierarchical voxel-level features, and a top-down multi-scale fusion network that aggregates geometric cues across different spatial scales. Here, the term ‘global’ denotes multi-scale aggregation rather than a conventional global attention mechanism.

Given the voxel-wise features, the backbone encoder refines these features through a sequence of residual bottleneck blocks. Each 3D bottleneck block follows the structure defined in Equation 19.

(19) $$\begin{array}{c} z1\;=\;\sigma \left(BN\left(conv1\times 1\times 1\left(V\right)\right)\right)\\ \text{z}2\;=\;\sigma \left(BN\left(conv3\times 3\times 3\left(z1\right)\right)\right)\\ \begin{array}{c} \text{z}3\;=\;BN\left(conv1\times 1\times 1\left(z2\right)\right)\\ Bottleneck\left(V\right)=\sigma \left(z_{3}+down\left(V\right)\right) \end{array} \end{array}$$

To produce hierarchical 3D features, multiple bottlenecks are grouped into three stages with spatial strides: $V_{1}=f_{1}\left(V_{0}\right),\;V_{2}=f_{2}\left(V_{1}\right),V_{3}=f_{3}\left(V_{2}\right)$. Here, the multi-scale geometric features are defined as $V^{3D}=\left\{F_{0},F_{1},F_{2}\right\}=\left\{V_{0},V_{1},V_{2}\right\}$, where *F*_0_ captures fine-grained spatial detail, *F*_1_ provides structural mid-level cues, and *F*_2_ represents context-rich geometric abstraction.

The neck first constructs lateral feature streams *L_l_* = *F_l_*. A top–down pathway then aggregates high-level geometry cues by upsampling and concatenating features. Specifically, the upsampled features ${\tilde{L}}_{l}=Up_{3D}\left(P_{l+1}\right)$ are fused with the lateral features to form $C_{l}=Concat\left(L_{l},{\tilde{L}}_{l}\right)$. This aggregated representation is further refined through two convolutional stages. This aggregated representation is further refined through two convolutional stages, as defined in Equations 20 and 21.

(20) $$U_{l}=\emptyset_{l}^{\left(1\right)}\left(C_{l}\right)=Conv_{1\times 1\times 1}\left(C_{l}\right)$$

(21) $$P_{l}=\emptyset_{l}^{\left(3\right)}\left(U_{l}\right)=Conv_{1\times 1\times 1}\left(U_{l}\right)$$

### 2.4 Training loss

To explicitly incorporate global SSC performance into the network optimization, we build upon the 2D binary affinity loss (Li et al., 2023) and introduce a multi-class version that directly optimizes scene-wise and class- wise metrics.

Specifically, we optimize the class-wise derivable precision, and specificity where *P_c_* and *R_c_* measure the performance of similar class c voxels, and *S_c_* measures the performance of dissimilar voxels (i.e. not of class c). Let *p_i_* be the ground truth class of voxel *i*, and ${\hat{p}}_{i,c}$ be its predicted probability to be of class *c*. These class-wise metrics are defined in Equation 22.

(22) $$\begin{array}{c} P_{c}\left(\hat{p},p\right)=log\frac{\sum_{i}{\hat{p}}_{i,c}〚p_{i}=c〛}{\sum_{i}{\hat{p}}_{i,c}}\\ R_{c}\left(\hat{p},p\right)=log\frac{\sum_{i}{\hat{p}}_{i,c}〚p_{i}=c〛}{\sum_{i}〚p_{i}=c〛}\\ S_{c}\left(\hat{p},p\right)=log\frac{\sum_{i}\left(1-{\hat{p}}_{i,c}\right)\left(1-〚p_{i}=c〛\right)}{\sum_{i}\left(1-〚p_{i}=c〛\right)} \end{array}$$

With 〚·〛 denoting the Iverson brackets. For more generality, our loss ${ℒ}_{scal}$ maximizes the above class-wise metrics, as defined in Equation 23.

(23) $${ℒ}_{scal}\left(p,p\right)=-\frac{1}{C}\sum_{c=1}^{C}\left(P_{c}\left(\hat{p},p\right)+R_{c}\left(\hat{p},p\right)+S_{c}\left(\hat{p},p\right)\right)$$

In practice, we optimize semantics ${ℒ}_{scal}^{sem}={ℒ}_{scal}\left(\hat{y},y\right)$ and geometry, where $\left\{y,y^{geo}\right\}$ are semantic and geometric labels with respective predictions $\left\{\hat{y},{\hat{y}}^{geo}\right\}$.

Training is driven by a multi-term objective. We first adopt a cross-entropy loss ${ℒ}_{ce}$ with class-frequency reweighting to mitigate class imbalance. To better align optimization with the evaluation criteria, two affinity losses, ${ℒ}_{scal}^{geo}$ and ${ℒ}_{scal}^{sem}$, are further included, targeting geometric IoU at the scene level and semantic mIoU at the class level. Depth estimation is supervised by an explicit term, which constrains the depth network through its predicted depth-probability distribution. The final objective (Tseng et al., 2025) is therefore expressed as Equation 24.

(24) $$L=\lambda_{d}{ℒ}_{d}+\lambda_{c}{ℒ}_{ce}+\lambda_{g}{ℒ}_{scal}^{geo}+\lambda_{s}{ℒ}_{scal}^{sem}$$

Where ${ℒ}_{d}$ denotes the depth estimation loss. In practice, we set $\lambda_{d}=0.1$, $\lambda_{c}=1.0$, $\lambda_{g}=1.0$, and $\lambda_{s}=1.0$, the depth loss is down-weighted to prevent it from dominating the optimization.

### 2.5 Task-oriented structural mapping head

While the proposed monocular SSC framework produces dense voxel-wise semantic occupancy prediction, downstream agricultural applications require task-oriented structural representations rather than raw voxel grids. To bridge voxel-level perception and actionable agronomic or navigation tasks, we introduce a lightweight Task-Oriented Structural Map module built upon the SSC output.

Given the predicted occupancy probability $P_{occ}\left(x,y,z\right)$ and semantic distribution, we derive three structural maps: height map, volume map, and obstacle map.

#### 2.5.1 Height map

Let $P_{occ}\left(x,y,z\right)$ denote the predicted occupancy probability at voxel (*x*, *y*, *z*), and let $P_{sem}^{plant}\left(x,y,z\right)$ denote the semantic probability of the plant class. To isolate crop structures while excluding non-relevant objects (e.g., ground, pots, structures), plant occupancy is defined as Equation 25.

(25) $$O_{p}\left(x,y,z\right)=\prod \left(P_{occ}>\tau_{occ}\right)\wedge \prod \left(P_{sem}^{plant}>\tau_{sem}\right)$$

The canopy height is computed as Equation 26.

(26) $$H\left(x,y\right)=max\left\{z|O_{p}\left(x,y,z\right)=1\right\}\cdot \gamma$$

where γ is the voxel resolution. This height map captures the vertical crop structure and supports agronomic analysis.

#### 2.5.2 Volume map

Local canopy density is obtained by column-wise voxel accumulation, as defined in Equation 27.

(27) $$V_{layer}\left(x,y\right)=\sum_{Z}O_{p}\left(x,y,z\right)\cdot \gamma^{3}$$

This map reflects canopy thickness and spatial distribution. This layered representation enables finer-grained analysis of canopy densification patterns.

#### 2.5.3 Obstacle and traversability map

To incorporate semantically dangerous objects (e.g., trunks, poles, humans), a risk-aware score is defined as Equation 28.

(28) $$R\left(x,y,z\right)=w_{o}P_{occ}\left(x,y,z\right)+\sum_{c\in C_{h}}w_{c}P_{sem}\left(c,x,y,z\right)$$

The risk-aware occupancy is Equation 29.

(29) $$O^{\prime }\left(x,y,z\right)=I\left(R\left(x,y,z\right)>\tau_{risk}\right)$$

Obstacles within the robot’s vertical corridor are projected onto the ground plane, as defined in Equation 30.

(30) $$B\left(x,y\right)=\overset{z_{2}}{\underset{z=z_{1}}{\vee }}O^{\prime }\left(x,y,z\right)$$

After morphological dilation to account for the robot footprint, the final traversability map is T(x,y)=1−B(x,y). Optionally, a distance-based cost map can be derived via Euclidean distance transform for smooth motion planning.

## 3 Results

### 3.1 Datasets and experimental setup

#### 3.1.1 Semantic-KITTI dataset

To evaluate the general semantic scene completion capabilities of the proposed framework, we first conducted experiments on the Semantic-KITTI dataset. Semantic-KITTI is a large-scale outdoor autonomous-driving benchmark derived from the KITTI odometry dataset. It contains 22 sequences, among which sequences 00–10 provide voxel-wise semantic annotations. Following the standard validation protocol, sequence 08 was used for validation, and the remaining annotated sequences were used for training. Semantic-KITTI contains 20 voxel categories in total, including 19 semantic classes and one geometry occupancy class. This benchmark was used to evaluate whether the proposed depth-aware SSC framework can maintain competitive performance under a standard public evaluation setting.

#### 3.1.2 Horticultural dataset and data acquisition setup

To evaluate monocular 3D structural mapping under realistic horticultural conditions, we constructed a dedicated horticultural SSC dataset following the data organization and annotation protocol of Semantic-KITTI. Data collection was carried out at the Fruit Tree Research Institute of Shanxi Agricultural University. The dataset covers representative horticultural scenes, including potted plants, plant-row corridors, mixed canopy heights, dense foliage, ground surfaces, and facility background structures.

**Figure 4:**
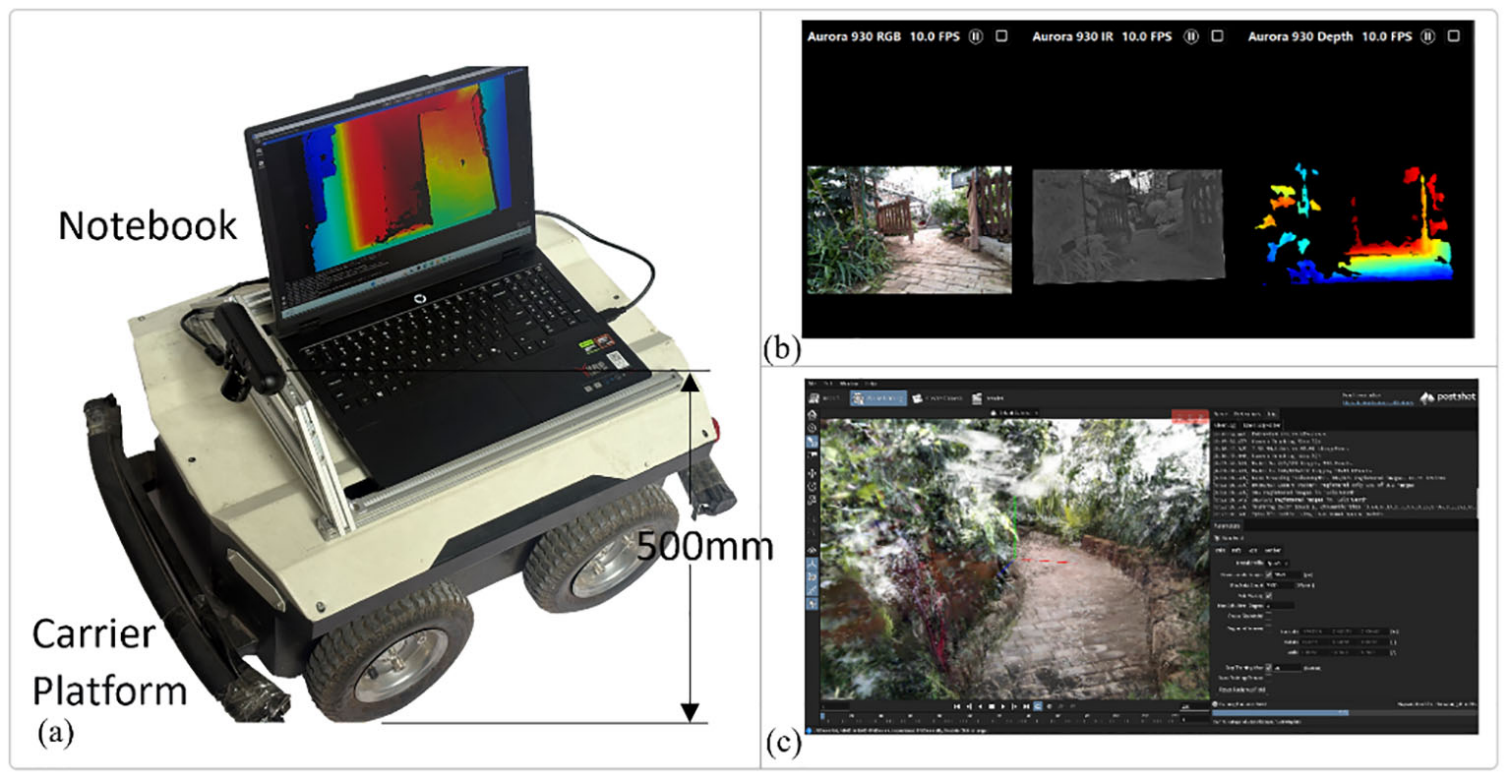
**(a)** Data acquisition equipment, **(b)** Depth camera data acquisition interface, **(c)** Jawest Postshot system.

Data acquisition was conducted using a low-speed mobile platform equipped with a Gemini336L structured-light camera, as shown in **Figure 4a**. The platform was designed to traverse agricultural scenes smoothly while capturing synchronized RGB images and depth maps. The data acquisition interface for RGB frames and depth observations is shown in **Figure 4b**. The platform moved along plant rows at approximately 0.5 m/s to maintain sufficient frame overlap and reduce motion blur. The camera was mounted at a height of approximately 0.5 m. During acquisition, synchronized RGB images and depth maps were collected. The image resolution was 1280 × 720 pixels, and the frame rate was 15 fps. The nominal depth accuracy of the camera was approximately 3 mm at 0.5 m and 7 mm at 1 m.

The use of the RGB-D camera in this stage is for dataset construction rather than for deployed inference. Since the proposed model is trained in a supervised manner, voxel-wise ground truth is required. The depth maps are therefore used to reconstruct local point clouds and support the generation of voxel occupancy labels, while the RGB images provide the corresponding monocular inputs. In the final inference stage, only the RGB image is fed into the trained network, and the depth stream is no longer required.

The dataset contains nine agricultural sequences in total. Following a sequence-based split protocol, sequences 00–07 were used for training, while sequence 08 was reserved exclusively for testing to ensure spatial independence between training and evaluation. Each sequence contains 260 frames, including RGB images, depth maps, semantic labels, voxel representations, and point clouds. Each accumulated point cloud contains approximately 340,899 points. Four semantic classes were defined for horticultural SSC: Road, Tree, Plant, and Other.

The voxel resolution was set to 0.01 m. Depth values were converted using a depth scale factor of 1000.0 and were further discretized into 64 intervals for depth-aware learning. The voxel-wise SSC ground truth was represented using a 3D grid of size 256 × 256 × 32.

#### 3.1.3 Environmental conditions, calibration and label generation

Data collection was carried out at the Fruit Tree Research Institute of Shanxi Agricultural University. Four representative acquisition environments were recorded during data collection. The measured temperatures were 22.9 °C, 21.0 °C, 25.4 °C, and 25.4 °C; the corresponding relative humidity values were 23.0%, 37.7%, 23.2%, and 30.9%, the illumination levels were 1900, 2900, 1900, and 3300 lux, respectively.

The collected scenes included low-canopy potted plants, mixed flowering and upright potted plants, and tall sparse leafy plants. These scenes contain common horticultural challenges such as canopy occlusion, irregular plant boundaries, mixed plant heights, reflective facility materials, and local illumination variations.

The Gemini336L camera provides factory-calibrated intrinsic and extrinsic parameters. The RGB and depth streams were automatically aligned during data acquisition using the camera SDK. The camera intrinsic matrix was obtained from the built-in calibration parameters of the Gemini336L camera. For the depth stream, the resolution was 1280 × 720, and the intrinsic parameters were set as $f_{x}=620.0$, $f_{y}=620.0$, $c_{x}=640.0$, and $c_{y}=360.0$. The corresponding intrinsic matrix was defined as Equation 31.

(31) $$K=\left[\begin{array}{ccc} 620.0 & 0 & 640.0\\ 0 & 620.0 & 360.0\\ 0 & 0 & 1 \end{array}\right]$$

The RGB-D sensor serves as an annotation and supervision tool during dataset construction, whereas the learned model performs monocular RGB-to-3D prediction during inference. Camera trajectories were estimated offline using the Jawest Postshot system, as shown in **Figure 4c**. The estimated poses were used to transform frame-level point clouds into a unified global coordinate system. The registered multi-frame point clouds were accumulated to generate static scene-level PLY files for semantic annotation and voxel-label generation.

Semantic annotation was performed at the point-cloud level using LabelCloud. The annotation team consisted of three experienced annotators. During annotation, large geometric entities such as road regions and continuous canopy regions were first selected using 3D bounding boxes. Boundary regions and isolated points were then refined using the point-picking tool.

Voxel labels were generated from the accumulated annotated point clouds. To better preserve safety-critical rigid obstacles and target plant structures, a priority-based voxel assignment rule was adopted. The class priority was defined as Tree>Plant>Road>Other.

If the number of tree-class points within a voxel exceeded a predefined threshold, the voxel was assigned to the tree class. If no high-priority class exceeded the threshold, the voxel was assigned the semantic class with the largest number of points using majority voting.

To generate dense voxel ground truth, a bidirectional sliding-window accumulation strategy was used. For each current frame, point clouds from a temporal window centered on that frame were accumulated. Considering the low platform speed of approximately 0.5 m/s, 20 frames were used for local accumulation. This window size improved voxel density within the camera field of view while reducing the influence of slight non-rigid plant motion.

#### 3.1.4 Implementation details and reproducibility

All experiments were implemented in Python using PyTorch and trained on NVIDIA A6000 GPUs. Input RGB images were resized to 1280 × 720 pixels. EfficientNet-B7 was adopted as the image backbone, with a feature channel dimension of 64. The resulting 2D feature map has a spatial resolution of 32 × 32, which is lifted into a 3D voxel representation of size 128 × 128 × 16. For the horticultural dataset, valid depth values in the range of 0.1-20m are used for data generation.

The model was trained for 50 epochs using the AdamW optimizer with a learning rate of 1×10^-4^ and a batch size of 4. Semantic segmentation loss, depth estimation loss, and SSC loss are jointly optimized. To prevent the depth loss from dominating the optimization process, it is weighted by a factor of 0.1 throughout training.

#### 3.1.5 Evaluation metrics

For semantic scene completion, mean Intersection-over-Union (mIoU) was used as the main semantic evaluation metric. For each semantic class, IoU was computed using correctly predicted voxels, falsely assigned voxels, and missed ground-truth voxels. The final mIoU was obtained by averaging the IoU values over all semantic classes.

Given a semantic class, we compute $IoU_{c}=\frac{TP_{c}}{TP_{c}+FP_{c}+FN_{c}}$, with *TP_c_* the correctly predicted voxels of class, *FP_c_* the voxels incorrectly assigned to class, and *FN_c_* the missed voxels that belong to *c* in the ground truth. This score reflects how well predictions overlap with the ground truth for each class.

We additionally report Precision on the proposed horticultural dataset: precision=TP/(TP+FP). Precision reflects the reliability of predicted occupied or semantic voxels. A higher Precision indicates fewer false-positive structural predictions, which is important for obstacle-aware traversability mapping and safe robotic operation.

In addition, for task-oriented structural map evaluation, canopy height and canopy volume proxy are evaluated using Mean Absolute Error (MAE), Root Mean Square Error (RMSE), and Relative Error against manual field measurements. These complementary metrics provide a more complete assessment of voxel-level geometry completion, semantic consistency, prediction reliability, and physical structural measurement accuracy.

### 3.2 Quantitative results

#### 3.2.1 Comparison on semantic KITTI

To address benchmarking rigor, we compare the proposed method with representative and recent state-of-the-art monocular SSC methods, including MonoScene, OccFormer, VoxFormer, H2GFormer (Wang and Tong, 2024), SGFormer (Wu et al., 2024), H2GFormer (Wang and Tong, 2024), scanSSC (Bae et al., 2025), PPMNet (Lu et al., 2025), DISC (Liu et al., 2025a), and MARE (Tseng et al., 2025). These baselines cover projection-based, transformer-based, depth-aware, and geometry-aware SSC paradigms. For a fair comparison, we report standard occupancy IoU and semantic mIoU on the Semantic-KITTI validation set.

For our method, each experiment was conducted three times with different random seeds, and we report the mean ± standard deviation. For published baselines, we report the results from the original papers or their official implementations under the same evaluation protocol when available. As shown in **Table 1**, our method achieves an occupancy IoU of 46.52± 0.12%, outperforming DISC by 1.20 percentage points, corresponding to a relative improvement of 2.65%. Although the mIoU of our method is not the highest among all compared methods, it remains competitive. This is mainly because the proposed framework is designed to prioritize geometry completion and vegetation-related structural consistency, rather than fine-grained urban-driving categories. From the class-wise IoU results in **Table 1**, several of the best-performing categories are also those frequently observed in facility agriculture scenarios. For the vegetation class, our IoU reaches 28.21, outperforming SGFormer by 3.71 percentage points and ScanSSC by 3.01 percentage points. Achieving the best IoU on vegetation and terrain suggests that our model is well-suited to the requirements of agricultural scenes, particularly for constructing 3D semantic representations in controlled-environment agriculture. For the terrain class, our IoU is 39.58, exceeding the existing H2GFormer method by 3.32 percentage points.

**Table 1:** Quantitative comparison of semantic scene completion performance on the Semantic-KITTI set.

Method	IoU	Road	Sidewalk	Parking	Other-grnd	Building	Car	Truck	Bicycle	Motorcycle	Other-veh	Vegetation	Trunk	Terrain	Person	Bicyclist	Motorcyclist	Fence	Pole	mIoU
MonoScene	34.2	54.7	27.1	24.8	5.7	14.4	18.8	3.3	0.5	0.7	4.4	14.9	2.4	19.5	1.0	1.4	0.4	11.1	3.3	11.1
OccFormer	34.5	55.9	30.3	31.5	6.5	15.7	21.6	1.2	1.5	1.7	3.2	16.8	3.9	21.3	2.2	1.1	0.2	11.9	3.8	12.3
VoxFormer	44.2	53.6	26.5	19.7	0.4	19.5	26.5	7.3	2.0	0.6	7.8	26.1	6.1	33.1	1.9	2.0	--	7.31	9.2	13.4
scanSSC	44.5	66.2	35.9	35.1	12.5	25.3	27.1	3.5	3.5	3.2	6.1	25.2	11.0	30.6	1.8	5.3	0.7	20.5	8.4	17.4
PPMNet	31.8	56.5	28.3	29.9	10.2	14.2	19.6	4.2	3.4	3.4	4.6	15.2	3.3	21.1	3.2	3.4	0.6	11.9	2.2	12.6
SGN-S	41.9	57.8	29.2	27.7	5.2	23.9	24.9	2.7	0.4	0.3	4.0	24.2	10.0	25.8	1.1	2.5	0.3	14.2	7.4	14.0
H2G	44.7	57.0	29.4	21.7	0.3	20.5	28.2	6.8	0.1	0.9	9.3	27.4	7.8	36.3	1.2	0.1	--	8.0	9.9	14.3
NDC-Scene	37.2	59.2	28.2	21.4	1.7	14.9	26.3	14.8	1.7	2.4	7.7	19.1	3.5	31.0	3.6	2.7	--	6.7	4.5	12.7
TPVFormer	34.3	55.1	27.2	27.4	6.5	14.8	19.2	3.7	1.0	0.5	2.3	13.9	2.6	20.4	1.1	2.4	0.3	11.0	2.9	11.3
DISC	45.3	63.1	34.7	34.6	12.6	26.6	26.7	5.5	4.0	4.7	8.1	26.5	10.3	29.3	2.8	2.5	1.1	19.3	8.4	17.4
MARE	43.2	56.3	25.9	19.5	0.7	23.4	29.1	--	2.8	3.6	13.5	26.8	8.3	33.8	3.2	2.1	0.0	10.4	9.4	15.4
SGFormer	44.4	64.3	34.2	34.1	12.1	25.8	26.1	4.30	3.7	1.3	2.7	24.5	11.2	29.3	1.7	3.6	0.4	18.7	8.7	16.6
Our	46.5± 0.12	63.1	32.5	18.6	7.2	25.0	33.0	9.75	1.4	2.5	7.5	28.2	9.5	39.6	2.9	0.5	0.0	11.4	10.6	15.9± 0.16

These gains can be largely attributed to the proposed Depth-Aware Decoder Module, which performs explicit depth recovery and 2D-to-3D feature fusion during the view transformation stage. By further integrating geometric and semantic cues from both 2D and 3D spaces, DADM provides a stronger and more reliable input to the downstream 3D SSC module, enabling more accurate recovery of depth and voxel-wise semantic information for agriculture-related elements.

Since the Semantic-KITTI dataset lacks an open test set ground truth, it is common practice to evaluate the results on the validation set.

**Figure 5:**
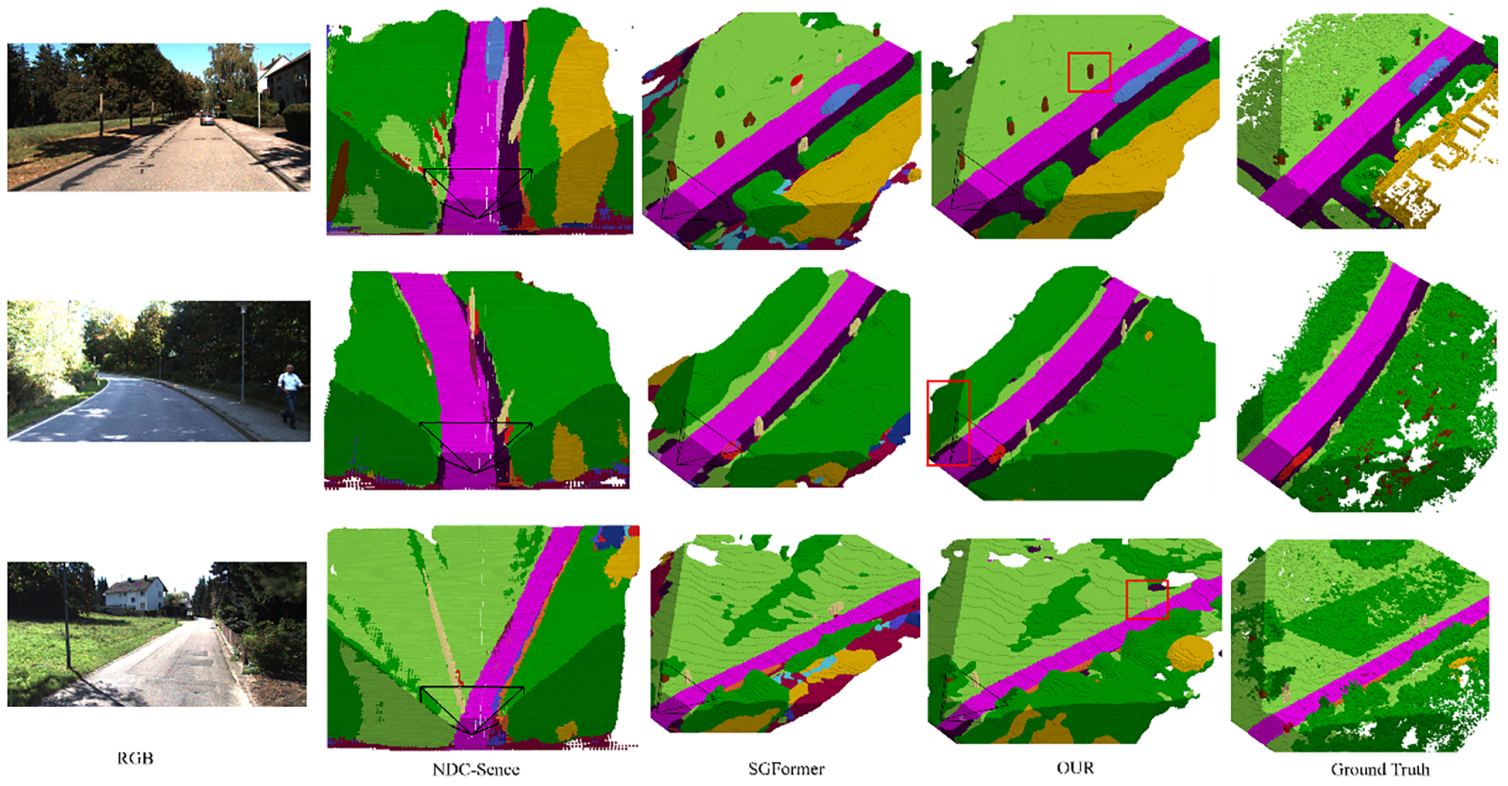
Qualitative comparison of semantic scene completion results on the Semantic-KITTI validation set. The columns show RGB input, NDC-Scene, SGFormer, Ours, and Ground Truth. Different colors indicate semantic categories following the Semantic-KITTI color mapping.

**Figure 5** presents qualitative visual comparisons on the Semantic-KITTI dataset. We selected scenes that are relatively more relevant to agricultural settings for visualization. In the first scene, compared with NDC-Scene, our method successfully reconstructs each tree more completely. SGFormer shows noticeable errors in predicting trees at long ranges, whereas our approach yields more stable and coherent far-field predictions. The third scene is similar to the first one but contains different roadside objects; as observed, our method produces more accurate and complete predictions for these roadside structures than the competing methods. Together, these two cases demonstrate that the proposed DADM, which performs explicit depth recovery and 2D-to-3D feature fusion during view transformation-effectively mitigates depth projection ambiguity.

In the second scene, both NDC-Scene and SGFormer also perform well, likely because the surrounding vegetation is dense and the scene provides strong geometric and semantic cues, thereby reducing ambiguity. Our method remains competitive in this scenario. Importantly, in the highlighted regions (red boxes), our results exhibit no obvious projection ambiguity: by leveraging geometrically consistent view transformation and voxel pooling to obtain voxel-grid-aligned 3D representations, our framework supports the global modeling of semantic context and geometric relations, leading to improved structural coherence in challenging areas.

Both quantitative and qualitative evaluations demonstrate the advantages of our method in agriculture-related scenes. The proposed approach effectively accommodates the high variability of agricultural environments and reduces the reliance on accurate pose measurements. Moreover, experiments on the Semantic-KITTI benchmark validate the effectiveness of our framework and provide strong empirical evidence to support its transfer and deployment on our in-house dataset.

#### 3.2.2 Comparison on our dataset

To further validate the effectiveness of our model, we constructed a horticultural dataset and conducted comparative experiments against representative baselines such as MonoScene and OccFormer. We also evaluated the generalization capability of the model through cross-scene validation, in which the model was trained on three scenes and tested on the remaining held-out scene.

The quantitative results are summarized in **Table 2**. To better accommodate the characteristics of agricultural environments, we first pretrained the model on Semantic-KITTI and used the resulting weights as initialization for our agricultural setting, followed by transfer learning on the proposed dataset. Our method achieves the best overall performance, with an 82.31% occupancy IoU and an 84.26% mIoU. Compared with SGFormer, the strongest baseline on this dataset, our method improves the occupancy IoU by 0.47 percentage points and the mIoU by 3.28 percentage points, corresponding to relative improvements of 0.57% and 4.05%, respectively. These gains demonstrate that the proposed depth-aware decoding and NCS-guided geometry encoding are highly effective in vegetation-dominant horticultural scenes characterized by weak pose accuracy and severe canopy occlusion.

**Table 2:** Quantitative comparison of semantic scene completion performance on our dataset.

Methods	Road	Tree	Plant	Other	IoU	Δ IoU/RI	mIoU	Δ mIoU/RI	Precision
MonoScene	54.78	87.63	75.74	68.34	78.37± 0.18	-3.47/-4.24	71.62± 0.26	-9.36/-11.56	47.83
OccFormer	52.54	86.34	88.54	68.92	77.65± 0.09	-4.19/-5.12	74.08± 0.11	-6.90/-8.52	60.97
NDC-Scene	68.87	84.23	86.43	77.75	80.56± 0.16	-1.28/-1.56	79.32± 0.23	-1.66/-2.05	70.42
DISC	72.68	87.92	89.84	77.84	81.50± 0.21	-0.34/-0.42	83.00± 0.14	+2.02/+2.49	--
SGFormer	70.23	89.34	90.12	74.21	81.84± 0.09	ref	80.98± 0.08	ref	84.32
Our	73.98	88.32	90.45	84.31	82.31± 0.06	+0.47/+0.57	84.26± 0.13	+3.28/+4.05	86.25

SGFormer is used as the reference baseline. Δ denotes the absolute performance difference between each method and SGFormer. Relative improvement (*RI*) is reported with respect to each baseline as $RI=\frac{\text{Method}-\text{ref}}{\text{ref}}\times 100\%$.

Both NDC-Scene and SGFormer introduce dedicated designs to reduce dependence on pose information, and consequently outperform the other baselines. In our dataset construction, we explicitly considered the characteristics of horticultural operations: data are captured using a structured-light camera, and the sensor pose is estimated from video streams. Since pose accuracy directly impacts 3D perception quality, mitigating pose dependence is crucial. To this end, DADM is designed to reduce reliance on pose parameters. The comparative results in **Table 2** indicate that DADM effectively meets the weak-pose-dependency requirement in agricultural scenarios, producing predictions that exhibit high overlap with ground-truth annotations in facility horticultural environments.

**Figure 6:**
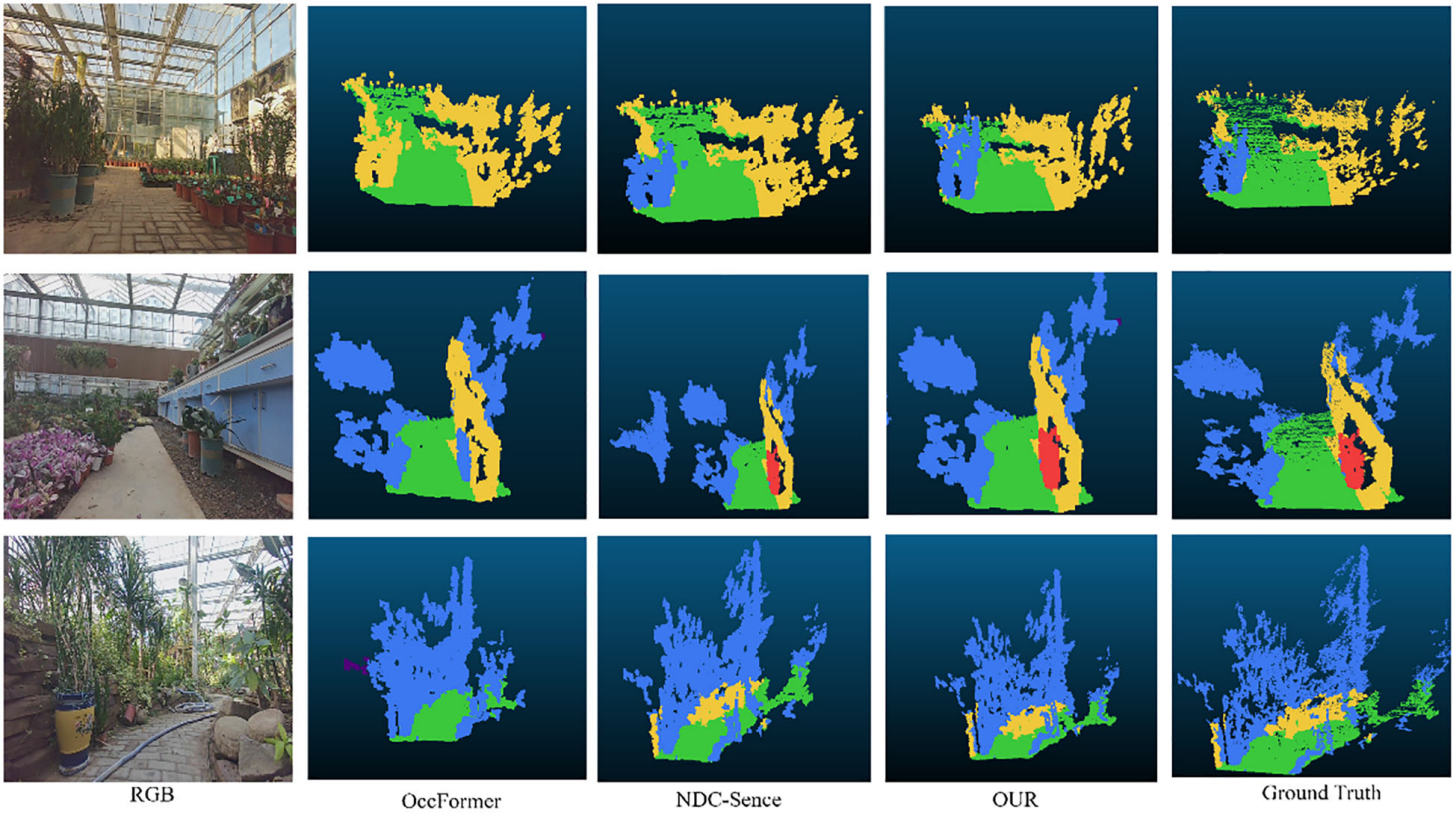
Qualitative comparison of semantic scene completion results on the proposed horticultural dataset. The columns show RGB input, OccFormer, NDC-Scene, Ours, and Ground Truth. Different colors represent Road, Tree, Plant, and other categories.

**Figure 6** presents qualitative visualizations on our dataset with comparisons among multiple methods. As observed, OccFormer and NDC-Scene exhibit noticeable depth projection ambiguity in facility agricultural environments, which is likely induced by errors in the estimated sensor pose. In contrast, our method produces predictions that are significantly closer to the ground truth, demonstrating stronger robustness under noisy or weak pose supervision.

### 3.3 ablation study

We conduct ablation studies on the Semantic-KITTI validation set to analyze the contributions of individual components within the proposed method. IoU and mIoU are adopted as the evaluation metrics.

The baseline model consists of a 2D encoder and a 3D decoder, where the projection from 2D image features to the 3D voxel space is implemented using the FLoSP algorithm. Based on this baseline, we progressively introduce additional components to construct different model variants: Model A incorporates a Global Encoder (GE) to enhance 3D feature representation through hierarchical voxel encoding. Model B further adds an Occupancy Head (Occ-Head) to generate 3D semantic prediction maps. Model C replaces the original FLoSP-based projection with the proposed DADM, which enables explicit depth recovery and joint fusion of 2D-to-3D branch features during view transformation. Finally, Model D integrates the proposed NGE, which explicitly embeds normalized depth from the NCS into voxel positional encoding, ensuring that all attention operations are performed under a geometrically consistent coordinate system.

As shown in **Table 3**, Model C achieves an occupancy IoU of 45.82% and improves geometric consistency in the vegetation and terrain categories, which are dominant in horticultural environments. These results indicate that DADM alleviates depth-projection ambiguity and benefits certain categories, yet the overall gain remains limited. This suggests that simply resolving projection ambiguity at the view transformation stage may be insufficient to consistently improve global completion quality.

**Table 3:** Ablation study of the architectural components on Semantic-KITTI set.

Method	GE	Occ-Head	DADM	NGE	IoU	mIoU	Improvement
Base					45.59	14.90	--
A	✓				45.22	15.59	-1.66/-1.97
B	✓	✓			45.76	15.45	-1.63/-2.88
C	✓	✓	✓		45.82	15.76	-1.46/-0.89
D	✓	✓	✓	✓	46.52	15.90	--

Motivated by this observation, we investigate whether depth cues can be more deeply integrated into the backend 3D decoding and reasoning process to enhance overall performance. Accordingly, we introduce the NCS-Guided Geometry Encoder, which explicitly injects normalized depth into voxel positional embeddings, enabling more effective spatial reasoning within the 3D voxel space. The final model achieves an occupancy IoU of 46.52 and an mIoU of 15.90, yielding the best overall performance among the ablated variants.

Using Semantic-KITTI as the base benchmark, these ablation studies provide a rigorous validation of our design choices and offer empirical support for deploying the proposed framework in agricultural scenarios. Moreover, each ablated component serves as a practical baseline for our in-house agricultural dataset construction and system configuration.

### 3.4 Task-level structural map evaluation

To quantitatively validate the reliability of the proposed task-oriented structural maps, we conducted a field-based measurement experiment. The algorithm was deployed on a Lenovo R7000P laptop, and all structural map computations were performed locally. The hardware configuration was as follows: an AMD Ryzen 9 8940HX CPU and an NVIDIA GeForce RTX 5060 Laptop GPU with 8 GB of VRAM. To ensure experimental consistency and reproducibility, the software environment, including the Python and PyTorch versions, was kept identical to that used on the training server.

#### 3.4.1 Measurement protocol and evaluation metrics

Manual Height Measurement. For each selected plant, the maximum canopy height was measured using a calibrated measuring ruler with millimeter precision. The height was defined as the vertical distance from the ground surface to the highest visible canopy point.

Manual Structural Volume Approximation. Direct physical measurement of true canopy volume is challenging in field conditions. Therefore, a geometric approximation method was adopted.

For each plant:

The maximum canopy height *H^gt^* was measured.

The horizontal canopy span (maximum lateral width) *W^gt^* was recorded.

For asymmetric plants, two orthogonal widths were measured and averaged.

The reference canopy volume *V^ref^* was approximated using a simplified geometric model $V^{ref}=\alpha \cdot H^{gt}\cdot {\left(W^{gt}\right)}^{2}$ where *α* is a shape coefficient determined according to canopy morphology. In practice, *α* was set to 0.21 for Set 1, which mainly contained compact low-canopy potted horticultural plants and was approximated by an ellipsoid-like canopy form. For Set 2, which consisted of mixed flowering and upright potted plants with moderately irregular canopy boundaries, *α* was set to 0.18. For Set 3, which contained tall sparse leafy potted plants with a more open canopy structure, *α* was set to 0.36. These coefficients were applied consistently to all plant-level samples within each set.

Let *H_i_* denote the predicted canopy height at sample, and let $H_{i}^{gt}$ denote the manually measured height. We define the mean absolute error (MAE) and root mean square error (RMSE) as Equations 32 and 33.

(32) $$\text{MAE}=\frac{1}{N}\sum_{i=1}^{N}|H_{i}-H_{i}^{gt}|$$

(33) $$\text{RMSE}=\sqrt{\frac{1}{N}\sum_{i=1}^{N}{\left(H_{i}-H_{i}^{gt}\right)}^{2}}$$

Let *V_i_* denote the predicted total canopy volume and let $V_{i}^{ref}$ denote the reference volume estimated from manual measurements. The relative error is defined as Equation 34.

(34) $$\text{Relative Error}=\frac{|V_{i}-V_{i}^{ref}|}{V_{i}^{ref}}$$

This section presents the results of measurements and calculations performed on plant-level samples. Potted plants were selected from horticultural scenes, and 20 plant-level samples were selected and manually measured to evaluate statistical reliability.

#### 3.4.2 Statistical reliability and uncertainty analysis

For each scene, the mean, MAE, RMSE, and 95% confidence interval were computed to quantitatively evaluate the consistency between the predicted structural maps and manual measurements.

As shown in **Table 4**, three representative plant structural types were selected for field measurement, including low-canopy potted horticultural plants, mixed flowering and upright potted plants, and tall sparse leafy potted plants. Canopy height estimation achieved MAE values of 0.019-0.026 m and RMSE values of 0.028-0.038 m across the three measurement sets. After incorporating statistical uncertainty, the absolute height error ranged from 0.019± 0.021m to 0.026± 0.028m, with 95% confidence intervals remaining at the centimeter level. For canopy volume estimation, the volume proxy achieved MAE values of 0.017-0.022m³ and relative errors of 8.4%-11.4%. These results indicate that the proposed structural maps are physically consistent and statistically reliable for practical horticultural measurement.

**Table 4:** Quantitative evaluation of the structural maps.

Set	Height			Volume				
	MAE	95% CI of MAE	RMSE	MAE	95% CI of MAE	RMSE	Relative error	
Set 1	0.022± 0.022	0.012–0.032	0.031	0.018± 0.019	0.010–0.026	0.026	8.7%
Set 2	0.026± 0.028	0.014–0.038	0.038	0.022 ± 0.022	0.012–0.032	0.031	11.4%
Set 3	0.019± 0.021	0.010–0.028	0.028	0.017 ± 0.018	0.009–0.025	0.025	8.4%
Overall	0.022 ± 0.024	--	0.033	0.019 ± 0.020	--	0.027	9.5%

*n* denotes the number of plant-level measurements in each measurement set. SD represents the standard deviation of absolute errors. The 95% confidence interval (CI) was estimated as $95\%\;CI=MAE\pm 1.96\times SD/\sqrt{n}$. Volume relative error was calculated using the manually approximated canopy volume as the reference.

**Figure 7:**
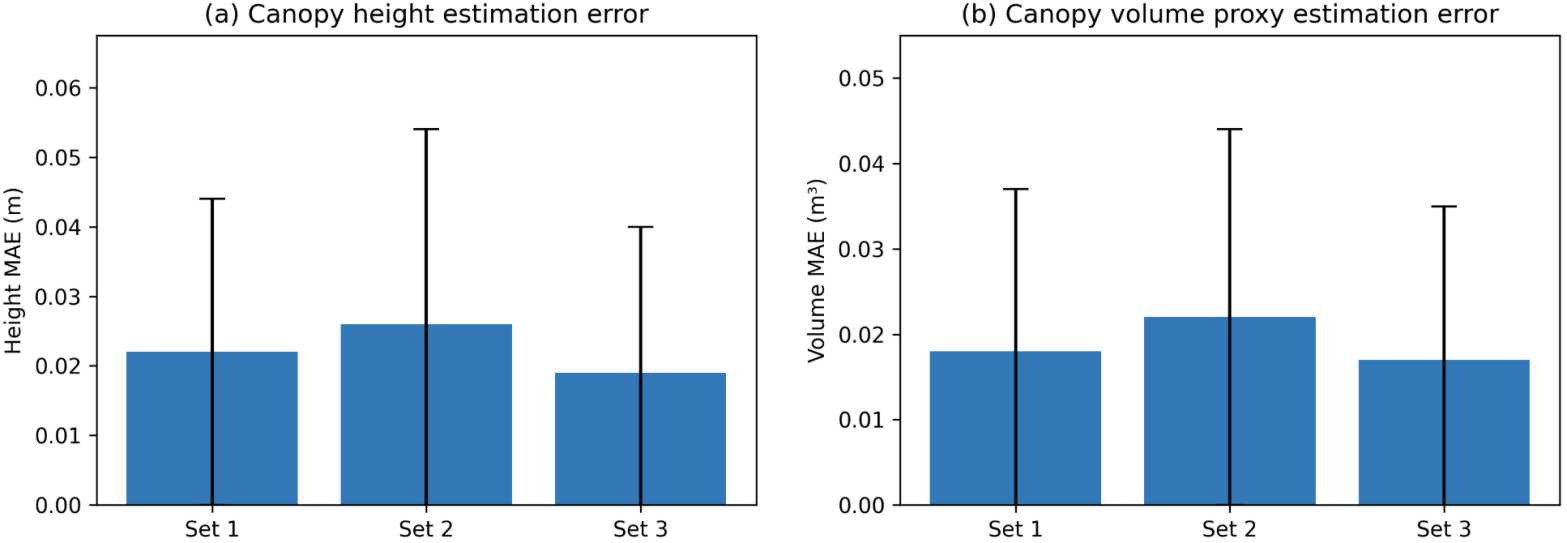
Error-bar analysis of canopy height and canopy volume proxy estimation. The vertical axes represent Height MAE (m) and Volume MAE (m³), respectively. Error bars indicate the standard deviation across plant-level measurements.

As shown in **Figure 7**, the error-bar results further illustrate the statistical reliability of the proposed structural maps across three representative plant structural types. For canopy height estimation, the MAE values were 0.022± 0.022 m, 0.026± 0.028 m, and 0.019± 0.021 m for Set 1, Set 2, and Set 3, respectively. For canopy volume estimation, the MAE values were 0.018± 0.019 m³, 0.022± 0.022 m³, and 0.017± 0.018 m³, respectively. Set 2 exhibited the largest average error and variability, suggesting that mixed flowering and upright potted plants are relatively more challenging for structural estimation.

#### 3.4.3 Deployment performance

**Figure 8:**
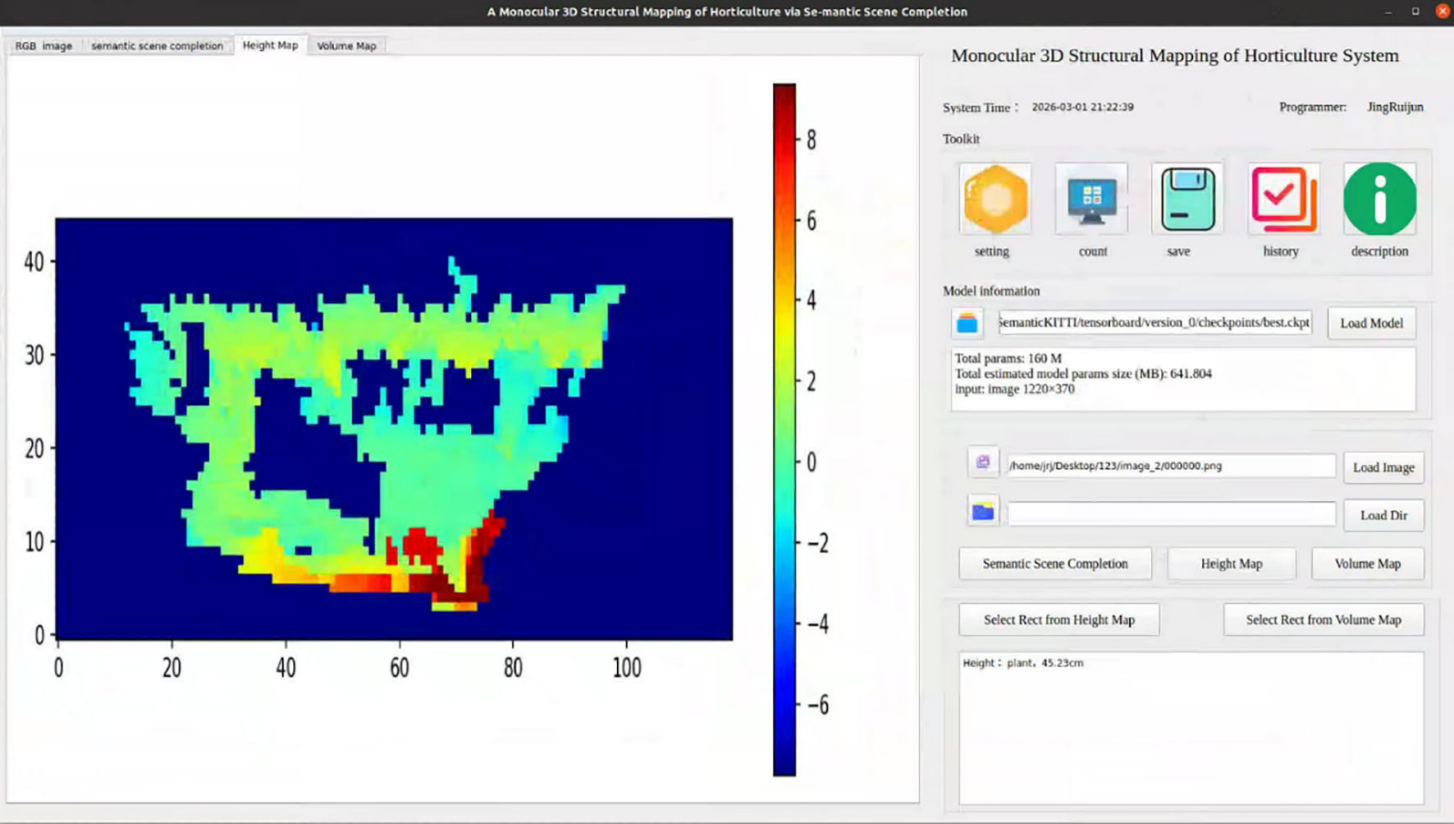
Deployment interface of the proposed structural mapping system. The interface displays the semantic scene completion result, height map, volume map, and selected plant-level measurements.

The system outputs are presented in **Figure 8**. The implemented framework supports the generation of Semantic Scene Completion (SSC) results, together with the corresponding Obstacle and Traversability Map, Height Map, and Volume Map.

An interactive selection mechanism was incorporated into the system interface, allowing users to define a region of interest using a bounding box to select specific vegetation instances. The height and volume of the selected plants were then computed automatically, with the calculation restricted to the target category within the selected region.

During execution, the peak GPU memory consumption was 6321 MB, and the average inference time for processing a single image was 0.529 s. These results indicate that the proposed framework can generate task-oriented structural maps with acceptable computational efficiency on a consumer-grade laptop, thereby supporting practical deployment in horticultural monitoring and agricultural robotic applications.

## 4 Discussion

We investigate monocular 3D structural mapping in horticultural environments through semantic scene completion (SSC). By using a single RGB camera, the proposed framework recovers dense voxel-wise occupancy and semantics and further derives task-oriented structural maps, including canopy height, a volume proxy, and obstacle-aware traversability. Compared with conventional monocular SSC methods that are primarily designed for urban or indoor semantic reconstruction, the proposed method aims to bridge the gap between voxel-level perception and actionable horticultural measurement.

The observed performance gains can be explained by the depth-aware design of the proposed framework. In horticultural scenes, leaves, stems, pots, and support structures frequently overlap in the image plane, which makes direct 2D-to-3D feature lifting prone to depth ambiguity and projection misalignment. The proposed Depth-Aware Decoder Module explicitly reconstructs depth-aware voxel representations through depth probability aggregation and progressive NCS-based voxel refinement. This approach provides a more geometrically consistent input for subsequent 3D reasoning. In addition, the NCS-Guided Geometry Encoder injects normalized depth cues into voxel positional embeddings, allowing attention operations to distinguish voxels with similar image coordinates but different depth positions. These mechanisms explain why the proposed method achieves stronger occupancy completion and improved structural consistency in vegetation-dominant scenes.

On Semantic-KITTI, the proposed method achieves an occupancy IoU of 46.52% and an mIoU of 15.90%. Although the mIoU is not the highest among all compared methods, the result is consistent with the design goal of this study. Semantic-KITTI contains many fine-grained urban categories, such as bicycles, motorcyclists, fences, and poles, which are not the primary targets of horticultural structural mapping. Our framework prioritizes geometry completion and vegetation-related structural consistency rather than optimizing all urban semantic categories equally. This explains why the method achieves the highest occupancy IoU and strong performance on agriculture-relevant classes such as vegetation and terrain, while maintaining a competitive but not best mIoU on the full urban benchmark. On the proposed horticultural dataset, the method achieves an occupancy IoU of 82.31% with an mIoU of 84.26%, improving the occupancy IoU by 0.47% percentage points and the mIoU by 3.28% percentage points over SGFormer, corresponding to relative gains of 0.57% and 4.05%, respectively. The larger gain on the horticultural dataset suggests that the proposed depth-aware representation is particularly beneficial for plant-dominant scenes with weak pose accuracy and severe canopy occlusion.

Compared with the urban Semantic-KITTI benchmark, which was collected for general autonomous-driving perception using a sensor-rich platform equipped with LiDAR, IMU, and calibrated sensors, our horticultural dataset is motivated by practical agricultural production, where sensing cost and field deployability are critical. Semantic-KITTI mainly describes structured urban scenes with rigid objects, road layouts, and clear geometric boundaries. In contrast, horticultural scenes contain highly repetitive crop-row textures, thin and deformable plant structures, leaf overlap, self-occlusion, greenhouse-related illumination changes, and visually similar plant instances. In our dataset, a structured-light RGB-D camera was used only to construct reliable voxel-level supervision, whereas the deployed model predicts 3D semantic structures from a single RGB image. This design targets low-cost autonomous agricultural operation, supporting not only robot navigation but also task-oriented 3D agronomic mapping. Moreover, slower and more constrained camera motion in crop rows can reduce motion blur, but it also limits multi-view geometric diversity and increases reliance on single-frame monocular reasoning, making real-time deployment more sensitive to repetitive textures, lighting variation, and occlusion.

Beyond voxel-level metrics, the task-oriented mapping head converts completed semantic voxels into interpretable structural layers without introducing additional trainable modules. The height map provides direct canopy height information, the volume proxy reflects local canopy density and spatial expansion, and the traversability map supports safety-aware robotic operations. Manual field measurements further validate the physical consistency of these structural maps. Across three measurement sets, canopy height estimation achieves MAE values of 0.019-0.026m and RMSE values of 0.028-0.038m, while the volume proxy achieves MAE values of 0.017-0.022m³ with relative errors of 8.4%-11.4%. The error-bar analysis further shows that the estimation uncertainty remains within an acceptable range. Set 2 exhibits slightly larger errors and variability, which is likely due to the mixed flowering and upright plant structures with more irregular canopy boundaries. Set 3 shows relatively lower errors, suggesting more stable structural estimation for sparse and vertically distinguishable leafy plants.

As shown in **Table 5**, compared with multi-sensor or multi-robot 3D mapping systems, which require complex calibration and hardware setups, the monocular design of our framework offers a low-cost, deployable solution that maintains high accuracy without relying on LiDAR or RGB-D sensors. Collectively, these features position our method as a practical advancement that bridges the gap between urban/indoor SSC models and real-world horticultural applications, addressing monocular depth ambiguity, weak-pose dependency, and the need for interpretable structural outputs.

**Table 5:** Benchmarking summary of sensing configuration and task-level capability.

Method	Input	Depth recovery	Weak-pose	Task-oriented maps	Low-cost
MonoScene	RGB	✗	✗	✗	✓
OccFormer	RGB	✗	✗	✗	✓
NDC-Scene	RGB	partial	✓	✗	✓
SGFormer	RGB	✗	partial	✗	✓
SLAM	RGB-D/LiDAR/RGB	✓	pose	partial	✗
Ours	RGB	✓	✓	✓	✓

Our framework uses RGB images as the input representation during inference. This choice is consistent with common camera outputs and reduces preprocessing complexity, but it may make the appearance features sensitive to illumination intensity, shadows, and exposure changes. Other color representations, such as HSV, may provide complementary chromatic cues by separating hue and saturation from brightness. Such representations could improve robustness in horticultural scenes with strong illumination variation or plant color differences. We did not introduce HSV-based input because the main objective is to validate RGB-only monocular 3D structural mapping, and HSV conversion does not provide additional depth or geometric measurements.

In terms of deployment, the proposed RGB-only configuration reduces sensing costs and system complexity compared with LiDAR-, RGB-D-, or multi-robot-based mapping systems. The system was deployed on a consumer-grade Lenovo R7000P laptop, with a peak GPU memory consumption of 6321MB and an average inference time of 0.529s per image. These results indicate that the framework is feasible for offline analysis, low-speed greenhouse monitoring, and field robotic applications with moderate real-time requirements. However, deployment on lightweight edge devices or high-speed mobile platforms still requires further optimization, such as adopting lightweight backbones, model pruning, quantization, temporal feature reuse, or resolution-adaptive inference. These directions will support broader scalability and practical use in precision horticulture.

## 5 Conclusions

We describe a low-cost monocular 3D structural mapping framework for horticultural environments via semantic scene completion (SSC). Using a single RGB camera, the proposed method reconstructs dense voxel-wise geometry and semantics and further derives task-oriented structural maps, including canopy height, a canopy volume proxy, and obstacle-aware traversability. This unified representation enables plant structural measurement and safe spatial reasoning without requiring LiDAR or RGB-D sensors.

Our proposed framework addresses monocular depth ambiguity through a Depth-Aware Decoder Module, which explicitly recovers depth-aware voxel representations and fuses 2D image features with 3D voxel features. In addition, the NCS-Guided Geometry Encoder injects normalized depth cues into voxel positional embeddings, enabling geometric-semantic reasoning in a depth-aware coordinate system. These designs improve geometric consistency and make the framework more suitable for vegetation-dominant horticultural scenes characterized by severe occlusion and limited pose accuracy.

Our method achieves an occupancy IoU of 46.52% on Semantic-KITTI, outperforming the strongest baseline DISC by 1.20 percentage points, corresponding to a 2.65% relative improvement. On the horticultural SSC dataset, the method achieves an occupancy IoU of 82.31% and an mIoU of 84.26%, outperforming SGFormer by 0.47 percentage points in occupancy IoU and 3.28 percentage points in mIoU, corresponding to relative gains of 0.57% and 4.05%, respectively. Field measurements further show that canopy height estimation achieves MAE values of 0.019-0.026 m and RMSE values of 0.028-0.038 m, while the canopy volume proxy estimation achieves MAE values of 0.017-0.022 m³ with relative errors of 8.4%-11.4%. The system also runs on a consumer-grade laptop with a peak GPU memory consumption of 6321 MB and an average inference time of 0.529 s per image.

This work provides a practical structural perception solution supporting canopy monitoring and safe robotic operation in horticultural environments. Specifically, we demonstrate that: First, monocular RGB-based SSC can provide a practical and scalable alternative to expensive 3D sensors for horticultural structural mapping. Second, depth-aware voxel reasoning significantly improves structural consistency in plant-dominant scenes. Third, task-oriented structural maps derived from semantic completion outputs can directly support canopy monitoring and safe robotic operation. Future work will focus on finer semantic granularity, such as separating leaves, flowers, and trellis structures, as well as on temporal modeling to improve stability under canopy motion, and facilitate long-term field deployment.

## Data Availability

The original contributions presented in the study are included in the article/supplementary material. Further inquiries can be directed to the corresponding author.
